# An Automated Aerosol Collection and Extraction System to Characterize Electronic Cigarette Aerosols

**DOI:** 10.3389/fchem.2021.764730

**Published:** 2021-11-04

**Authors:** Yeongkwon Son, Andrey Khlystov

**Affiliations:** Organic Analytical Laboratory, Division of Atmospheric Sciences, Desert Research Institute, Reno, NV, United States

**Keywords:** electronic cigarette, carbonyl, aldehyde, nicotine, testing, denuder, DNPH, public health

## Abstract

Electronic cigarette (e-cigarette) market increased by 122% during 2014–2020 and is expected to continue growing rapidly. Despite their popularity, e-cigarettes are known to emit dangerous levels of toxic compounds (e.g., carbonyls), but a lack of accurate and efficient testing methods is hindering the characterization of e-cigarette aerosols emitted by a wide variety of e-cigarette devices, e-liquids, and use patterns. The aim of this study is to fill this gap by developing an automated E-cigarette Aerosol Collection and Extraction System (E-ACES) consisting of a vaping machine and a collection/extraction system. The puffing system was designed to mimic e-cigarette use patterns (i.e., power output and puff topography) by means of a variable power-supply and a flow control system. The sampling system collects e-cigarette aerosols using a combination of glass wool and a continuously wetted denuder. After the collection stage, the system is automatically washed with absorbing and extracting liquids (e.g., methanol, an acetaldehyde-DNPH solution). The entire system is controlled by a computer. E-ACES performance was evaluated against conventional methods during measurements of nicotine and carbonyl emissions from a tank type e-cigarette. Nicotine levels measured using glass fiber filters and E-ACES were not significantly different: 201.2 ± 6.2 and 212.5 ± 17 μg/puff (*p* = 0.377), respectively. Differences in formaldehyde and acetaldehyde levels between filter-DNPH cartridges and the E-ACES were 14% (*p* = 0.057) and 13% (*p* = 0.380), respectively. The E-ACES showed reproducible nicotine and carbonyl testing results for the selected e-cigarette vaping conditions.

## Introduction

Popularity of electronic cigarettes (e-cigarettes) has been rapidly increasing, with sales per 4 weeks interval increasing from 7.7 million in 2014 to 17.1 million units in 2020 (Ali et al., 2020). Despite the popularity, e-cigarettes are known to emit potentially harmful compounds including heavy metals (Olmedo et al., 2018; Zhao et al., 2019), carbonyls (Geiss et al., 2016; Khlystov and Samburova 2016; Sleiman et al., 2016; Son et al., 2020), vaporized flavoring chemicals (Allen et al., 2015; Klager et al., 2017), and reactive oxygen species (ROS) (Lerner et al., 2015; Anderson et al., 2016; Son et al., 2019) in concentrations that could cause numerous adverse health impacts on respiratory, cardiovascular, neurological and immune system (Hua and Talbot, 2016; NASEM, 2018). In order to protect public health, the U.S. Food and Drug Administration (FDA) introduced a deeming rule regulating tobacco products including e-cigarette products requiring e-cigarette product testing and reporting potentially harmful substances (FDA, 2016).

In line with the regulatory efforts, scientists have been reporting a number of potentially harmful compounds in e-cigarette emissions. For instance, carbonyls are the most commonly reported and abundant harmful or potentially harmful compounds found in e-cigarette emissions (NASEM, 2018). However, there is a lack of standardized e-cigarette testing methods that can efficiently address a wide range of e-cigarette device settings (e.g., power output, coil type, and coil surface area, etc.), e-liquid compositions (e.g., base material, nicotine content, and flavoring, etc.), and vaping topography (i.e., puff duration, volume, and interval). For carbonyl measurements, most studies used either 2,4-dinitrophenyl-hydrazine (DNPH) cartridges (Goniewicz et al., 2014; Geiss et al., 2016; Khlystov and Samburova, 2016; Sleiman et al., 2016; Beauval et al., 2019) or impingers containing DNPH solution (Gillman et al., 2016; Flora et al., 2017; Farsalinos et al., 2018) to measure carbonyls in e-cigarette aerosol. While these methods are widely accepted, they are labor and cost intensive to allow quick and efficient testing of the rapidly evolving e-cigarette products under the wide range of use conditions. It is also worth mentioning that current commercially available smoking machines have been originally designed to target conventional cigarettes that do not produce large amounts of liquid particulates and large amounts of condensable gases that could hinder their performance.

The aim of this study was to develop a fully automated E-cigarette Aerosol Collection and Extraction System (E-ACES) which combines an e-cigarette vaping machine with an aerosol collection/extraction system. We developed and evaluated a prototype of E-ACES using a fourth generation “mod” type device filled with a tobacco flavored e-liquid. The performance of E-ACES for nicotine and carbonyl measurements was compared with conventional testing methods.

## Materials and Methods

### The E-Cigarette Aerosol Collection and Extraction System

**FIGURE 1:**
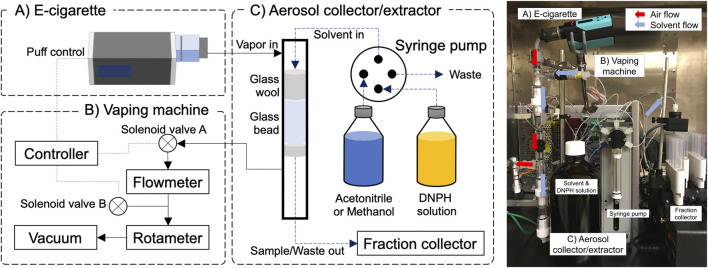
The E-cigarette aerosol collection and extraction system (E-ACES).

The E-ACES consists of an e-cigarette vaping machine and an aerosol collector/extractor (Figure 1). The vaping machine has two 24 V DC solenoid valves connected to a vacuum source. Air flow rates were monitored and controlled using a flowmeter (TSI, Shoreview, MN) and a rotameter. The solenoid valves, as well as an e-cigarette, were controlled using a U6 multifunction DAQ device with a PS12DC power switching board (LabJack Corporation, Lakewood, CO) connected to a laptop. To initiate a puff, the controller opened the solenoid valve A and closed the solenoid valve B (Figure 1), while simultaneously activating the e-cigarette using a relay channel for a “mod”-device or a power supply channel for a 510-thread type device. To terminate the puff, the controller de-activates the e-cigarette, closes the valve A and opens valve B. A Python script was used to operate the controller according to a pre-defined vaping topography.

### Sample Collection Using E-Cigarette Aerosol Collection and Extraction System

E-cigarette aerosol samples for nicotine and aldehyde analysis were collected using the E-ACES. A ‟mod” type e-cigarette device [ReuLeaux RX200 (WISMEC Electronics, Guangdong, China) and an Aspire Cleito atomizer (Shenzhen Eigate Technology, Shenzhen, China)] with a tobacco flavored e-liquid [3:7 = propylene glycol (PG): vegetable glycerin (VG), 6 mg/ml nicotine] at 50 W power output was used to test the E-ACES. We used 4 s puff duration, 100 ml puff volume, and 30 s puff interval based on the reported e-cigarette vaping topography (Dautzenberg and Bricard, 2015; Son et al., 2020). For the nicotine analysis, the aerosol collection part was rinsed with 5 ml methanol (LC/MS grade, Fisher Chemical, Waltham, MA) before aerosol generation. 5-puffs of e-cigarette aerosol were collected on the continuously wetted collector (methanol, 0.5 ml/min rate), then extracted three times with 2 ml methanol and the extract collected in the fraction collector. After that, the system was flushed with 5 ml methanol to assure no carry-over to the next analysis. Carbonyls in e-cigarette aerosols were collected as follows: 1) the collector was rinsed with 5 ml acetonitrile, 2) the glass wool and beads were soaked with 1 ml DNPH solution [22 mM DNPH (Spectrum, New Brunswick, NJ) in acetonitrile with 25 mM hydrochloric acid (Sigma-Aldrich, MO, United States), pH3-4], then 3) 5-puffs of e-cigarette aerosol were generated while the collector was continuously wetted with the DNPH solution (0.5 ml/min rate). After the collection step, the collector was extracted with 2 ml acetonitrile three times, then flushed with 5 ml acetonitrile. Extracted samples were stored in centrifuge tubes using the fraction collector. Nicotine and carbonyl sample collection and extraction were continuously done in triplicate (6 sampling cycles in total, 5 min sampling time per one condition) without any interruption (e.g., changing collection media, etc.). Collected sample volumes were consistent (less than 5% variation) across the samples.

### Sample Collection Using Conventional Testing Methods

For verification purposes, we also analyzed e-cigarette nicotine and carbonyl emissions using conventional methods. E-cigarette aerosols were generated using the vaping machine under the same vaping conditions (i.e., a “mod” device with tobacco flavored e-liquid, 50 W power output, 4 s puff duration, 100 ml volume, and 30 s interval). Nicotine samples were collected using glass fiber filter (GFF) pads (47 mm, MilliporeSigma, Burlington, MA). The sample filters were spiked with 40 μg of quinoline (98%, Sigma-Aldrich, MO, United States) as an internal standard. For carbonyl analysis, DNPH-coated glass fiber filters (ORBO 827, SUPELCO, CA, United States) followed by DNPH cartridges (Sep-Pak XPoSure Plus Short Cartridge, Waters, Milford, MA, United States) were used to assure collection of both particle- and gas-phase carbonyls (Son et al., 2020). All measurements were done in triplicate.

### Nicotine Analysis

The GFF were extracted with 4 ml methanol. 1 μl of the E-ACES extracts or the filter extracts were injected into an HPLC system (Waters 2,690 Alliance System with a model 996 photodiode array detector) equipped with an Agilent Polaris 3 column (C18-A, 3 μm, 100 × 2.0 mm). The mobile phase A was pH 7.9 phosphate buffer [8.5 mM Na_2_HPO_4_ (Electron Microscopy Sciences, PA, United States), 1.5 mM KH_2_PO_4_ (Beantown Chemical Corporation, NH, United States)] and the mobile phase B was methanol. The solvent gradient (0.1 ml/min flow rate) was 70% mobile phase A at 0-min and hold for 2.5 min, increase to 95% in 3.5 min, decease to 30% in 2 min and hold for 4 min, increase to 95% in 3 min and hold for 5 min, and decrease to 70% in 5 min and hold for 5 min. The total run time was 30 min. External standards of nicotine (99%, Sigma-Aldrich, MO, United States) and quinoline were prepared and quantified at 260 and 220 nm wavelengths, respectively. Limits of detection (LOD) and limits of quantification (LOQ) were estimated by adding three- and ten-times the standard deviation of seven measurements of the lowest calibration standard, respectively, to the mean blank sample value. LOD and LOQ for nicotine were 0.44 μg/ml and 1.47 μg/ml, respectively.

### Carbonyl Analysis

DNPH-filters and DNPH-cartridges were extracted with 2 ml of acetonitrile. The HPLC system described above were used to quantify carbonyl compounds. Sample injection and mobile phase flow rates were 2 μl and 0.25 ml/min, respectively. Acetonitrile (mobile phase A) and ultrapure water (mobile phase B) was used to separate carbonyl compounds. Mobile phase gradients were 42% phase A at 0 min and hold for 9 min, increase to 55% in 7 min and hold for 2 min, increase to 90% in 1 min and hold for 6 min, decrease to 42% in 1 min and hold for 4 min. Carbonyls (i.e., formaldehyde, acetaldehyde, acrolein, propionaldehyde, 2-butanone, benzaldehyde, glyoxal, and hexaldehyde) were detected at 360 nm wavelength and full spectrum readings (210–400 nm) were used to confirm individual compounds. A certified carbonyl calibration mixture (AccuStandard, CT, United States) was used to generate calibration curves. LOD and LOQ for the eight carbonyl compounds were estimated using the same method described above and ranged from 0.011 to 0.022 μg/ml and from 0.037 to 0.074 μg/ml, respectively.

### Statistical Analysis

Two-tailed Student’s t-tests were conducted to compare nicotine and carbonyl emission levels measured with the conventional methods and the E-ACES using the R software package version 3.4.3 (R Development Core Team, Vienna, Austria). Significances were determined at *p* = 0.05.

## Results

**FIGURE 2:**
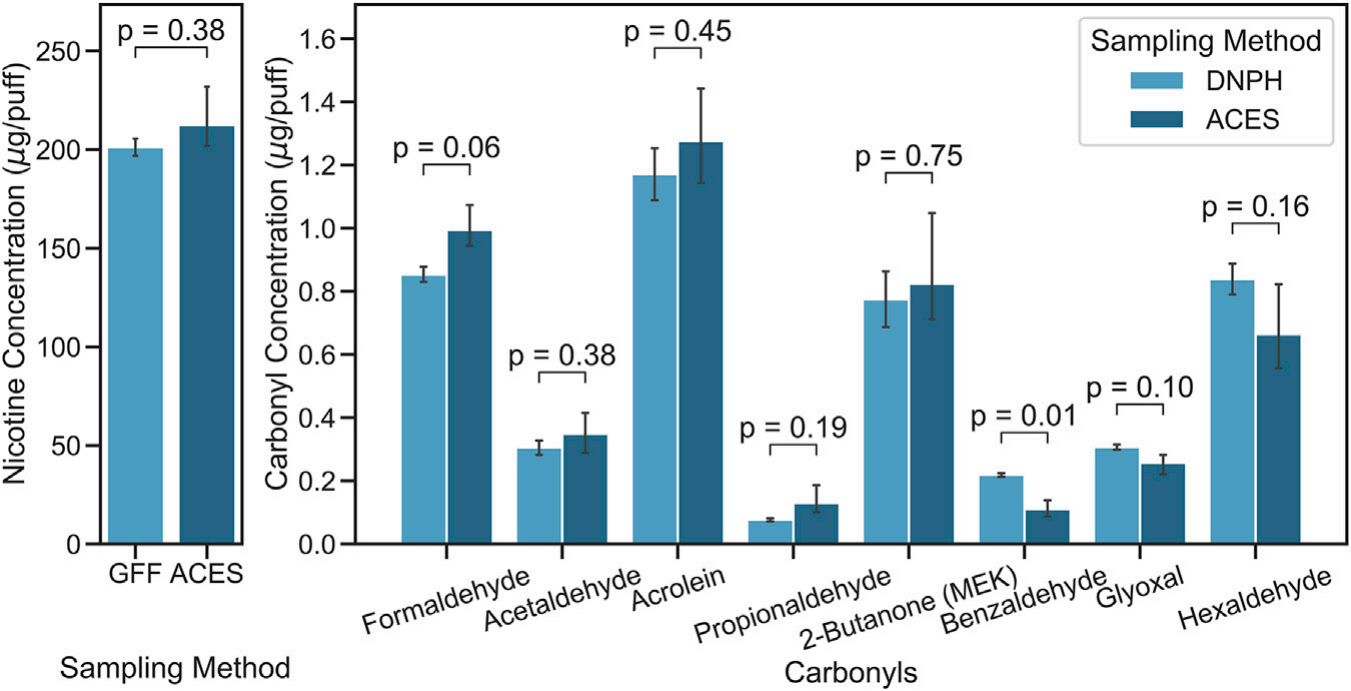
E-cigarette nicotine and carbonyl emission levels measured using the conventional methods [glass fiber filter (GFF) or DNPH-filer/cartridge (DNPH)] and the E-cigarette Aerosol Collection and Extraction System (E-ACES).

Figure 2 shows nicotine and carbonyl levels emitted from a “mod” type e-cigarette device. Measured nicotine levels were not significantly different, with values of 201.2 ± 6.2 and 212.5 ± 17 μg/puff (*p* = 0.377) for the conventional method (i.e., GFF method) and the E-ACES, respectively. Carbonyl emission levels measured using the DNPH-filter/cartridge method and the E-ACES were not significantly different except benzaldehyde. Formaldehyde and acetaldehyde, which are known carcinogens, were 0.854 ± 0.034 and 0.305 ± 0.031 μg/puff for DNPH-filter/cartridge method and 0.995 ± 0.069 and 0.350 ± 0.064 μg/puff for the E-ACES method, respectively (*p*-values > 0.057). Acrolein, propionaldehyde, and 2-butanone levels measured using the E-ACES method were slightly higher than the conventional method without significance (*p*-values > 0.193). Benzaldehyde levels determined using the conventional method were significantly higher than the E-ACES method (0.219 ± 0.008 μg/puff vs. 0.111 ± 0.026 μg/puff, *p* = 0.011). Conventional method could capture higher levels of glyoxal and hexaldehyde from e-cigarette aerosol than the E-ACES method (*p*-values > 0.102). Variabilities between the two methods were 5.6, 14.2, and 12.7% for nicotine, formaldehyde, and acetaldehyde, respectively.

## Discussion

We developed the fully automated E-cigarette Aerosol Collection and Extraction System (E-ACES) to characterize two types of e-cigarette emissions (i.e., nicotine and carbonyls). The results of the study show that the E-ACES method provides measurements that are in a good agreement with the conventional methods for nicotine and most of the measured carbonyl compounds. A low variability (< 15%) between the E-ACES and the conventional methods for nicotine and the two main toxic aldehyde (formaldehyde and acetaldehyde) levels provides confidence in the reliability of the developed system. The E-ACES system detection limit (LOD) for nicotine was 0.088 μg/puff and for aldehydes it was 0.016–0.244 μg/puff (formaldehyde and acetaldehyde were 0.016 and 0.032 μg/puff, respectively) using a 5-puff aerosol collection (4 s puff duration and 30 s interval) and 6 ml extraction. The system LOD is sufficiently low to detect nicotine and aldehydes emitted from most e-cigarette devices (Khlystov and Samburova, 2016; El-Hellani et al., 2018; Beauval et al., 2019; Gillman et al., 2020) except for acetaldehyde and glyoxal from a “pod” device (i.e., JUUL) (Son et al., 2020). The high sensitivity of the E-ACES system could allow cost and labor efficient sample collection (e.g., 5 min sampling/condition) for most e-cigarette products. The system sensitivity could be further improved by increasing the number of collected puffs. For instance, the system LOD for acetaldehyde will be 0.009 μg/puff if 35 puffs are collected (15 min sampling/condition), which will be sufficient for detection of acetaldehyde in JUUL aerosols containing 0.01 ± 0.01 μg acetaldehyde/puff (Son et al., 2020). A modular construction (vaping machine and aerosol collector/extractor) of the E-ACES is one of its strengths. The vaping machine could be applied to other sampling devices and/or methods. The aerosol collector/extractor could be applied to other tobacco products or even air pollution research to collect and extract samples automatically.

To the best of our knowledge, there is no such automated system for e-cigarette emission testing in a high-throughput format. Most studies reported so far have been using labor intensive sampling methods employing filters, cartridges, or impingers (Goniewicz et al., 2012; Uchiyama et al., 2013; Geiss et al., 2016; Khlystov and Samburova, 2016; Flora et al., 2017; Farsalinos et al., 2018; Qu et al., 2018; Son et al., 2018; Gillman et al., 2020). Havel et al. (2017) developed an e-cigarette-specific vaping machine that consists of a solenoid relay and a valve to generate e-cigarette aerosols. The vaping machine could operate e-cigarettes under a wide variety of vaping conditions. However, their sample collection and extraction were still manual and fairly labor intensive, involving three impingers containing 40 ml HCl solution, to test nicotine, propylene glycol (PG), and vegetable glycerin (VG). In another study, a direct e-cigarette aerosol collection method was evaluated (Olmedo et al., 2016). Strength of that collection method is that the system could collect undiluted e-cigarette aerosol samples, but the method needs long sampling times (15–20 min) and post sample processing steps for chemical analysis. Unlike these methods, the E-ACES automatically activates and puffs e-cigarettes, as well as collects and extracts e-cigarette aerosol samples. It also provides an opportunity to be interfaced with an online analytical instrument, thus providing a fully automated sample collection, extraction and analysis system.

The developed E-ACES prototype still has room for improvement. First, the E-ACES vaping machine needs a more sophisticated flow rate controller, such as a programable mass flow controller. The current prototype system employed a flow meter and a rotameter to control air flows through the system. The manual flow rate controller cannot address flow rate variations due to the pressure drop across the sample collection system. Even though we didn’t observe a significant pressure drop during testing, accurate and consistent flow rate would be desired because flow rate could change e-cigarette chemical emissions (Zhao et al., 2016; Son et al., 2020).

Second, particle collection efficiency of the aerosol collector needs to be improved. The glass wool plug proved to be sufficiently efficient to collect particles emitted from the “mod” type e-cigarette. However, the glass wool plug showed particle breakthrough for a “pod” type device (e.g., JUUL). This is because the “pod” type device generates smaller particles than the “mod” type device due to the lower power output (Floyd et al., 2018), which the glass wool could not capture. The glass wool filter could be replaced with a finer pore material such as a fritted glass in-line column filter.

Third, the collection efficiency could be further improved by optimization of derivatization and/or sampling methods. Our results showed that the E-ACES could collect similar or slightly higher levels of low-molecular carbonyls (e.g., formaldehyde and acetaldehyde), but lower amounts of large-molecular compounds (e.g., benzaldehyde and hexaldehyde). This could be due to differences in chemical properties of the target analytes. For instance, formaldehyde and acetaldehyde have high solubility (i.e., 13.2 M and 22.7 M, respectively) that could increase absorption rate, and thus their collection efficiency, while benzaldehyde has a low solubility (i.e., 0.06 M) and could be less efficiently absorbed during the contact time with DNPH (i.e., 20–40 min) (de Andrade and Tanner, 1992). Materials improving chemical sorption (e.g., silica gel beads) could help to capture chemicals emitted from e-cigarettes (Uchiyama et al., 2010). Collection efficiency of the DNPH solution-wetted filter/denuder type sampler could also be affected by pH, humidity, and characteristics of target carbonyl compounds (Kallinger and Niessner, 1997; John et al., 2020). Reaction between DNPH and carbonyl compounds are more efficient at acidic conditions than at neutral pH (Bicking et al., 1988). Our continuously wetting system supplying fresh DNPH solution to the filter/denuder minimizes pH changes over time, but the impacts of nicotine (pKa = 8.0) and other e-liquid constituents on pH need to be studied. Humid conditions (60% relative humidity [RH]) could accelerate DNPH-carbonyl derivatization reactions relative to dry conditions (0% RH) (John et al., 2020). E-liquids are known to contain different levels of water (Crenshaw et al., 2016). The impact of e-liquid water content should be evaluated to optimize the carbonyl collection in our system.

Lastly, there is an emerging need of testing metallic nanoparticles in e-cigarette aerosols due to their health risks (Mikheev et al., 2016; Olmedo et al., 2018; Wilson et al., 2019). The E-ACES system was originally designed to test e-cigarette chemical compound emissions (e.g., nicotine and carbonyls), but the developed system can be potentially adapted to measuring the aerosol metal content by using nitric acid as an extraction solution. Such an adaptation will require additional testing to characterize metal particle collection and extraction efficiency.

A limitation of this work is the limited number of e-cigarette devices and vaping conditions tested. A testing method and/or instrument should be evaluated systematically to prove their reliability. We have tested a “mod” type e-cigarette device with a tobacco flavored e-liquid under a single vaping topography (4 s puff duration, 100 ml volume, and 30 s interval). The tested e-cigarette is one of the most popular devices (i.e., “mod” and “pod” type device) and we used a vaping topography mimicking the common use patterns (Dautzenberg and Bricard, 2015; Robinson et al., 2015). This study aimed to demonstrate the capabilities of the new E-ACES method. We plan to further improve the E-ACES and evaluate it using different devices, e-liquids, and vaping conditions.

In conclusion, the E-ACES was developed to improve our ability to test for potentially harmful chemicals in e-cigarette aerosols that is critical for understanding the potential risks of e-cigarette use. Despite the limitations stated above, the E-ACES showed a good agreement with the conventional methods in measuring nicotine and carbonyls in e-cigarette aerosols. The developed instrument could benefit public health and tobacco regulatory science by accurately and rapidly testing a large variety of e-cigarette devices and e-liquids under different conditions.

## Data Availability

The original contributions presented in the study are included in the article, further inquiries can be directed to the corresponding author.
